# Public first aid education model design study based on user experience

**DOI:** 10.3389/fpubh.2023.1286250

**Published:** 2023-12-18

**Authors:** Jing Luo, Kaiqiao Zheng, Wudi Hong

**Affiliations:** College of Art and Design, Division of Arts, Shenzhen University, Shenzhen, Guangdong, China

**Keywords:** first aid, public educational model, user experience, emotion, CPR

## Abstract

**Background:**

Presently, China’s first aid penetration rate remains relatively low, leaving ample room for improvement in the existing first aid education model. Given its role as a service for the general public, public first aid education must thoroughly consider the learning needs and experiences of the public when designing the teaching mode.

**Methods:**

Semi-structured interviews were employed to gather detailed insights into participants’ experiences in the first aid learning process. Subsequently, NVivo was utilized to analyze the interviews and identify specific design strategies. Additionally, a 7-point scale questionnaire was employed to assess the intervention effects of music familiarity and the simulation of teaching aids on users’ willingness and confidence in learning. Building upon the design strategy, a “feedback device + app” approach was proposed. Finally, user satisfaction was evaluated through a scale questionnaire.

**Results:**

The use of familiar music had a significant positive effect on participants’ willingness and confidence to learn, while users’ fear of teaching aids had no effect on willingness and confidence. The user experience-based first aid education model can better meet the public’s learning needs for first aid knowledge and skills.

**Conclusion:**

This study proposes a first aid education model based on user experience design methodology, which optimizes the public’s self-learning experience by evoking positive emotions while circumventing negative emotions. The educational model was recognized by users in terms of design concepts and is expected to help increase first aid prevalence in the future.

## 1 Introduction

First aid is the immediate response to emergency accidents, involving prompt action from the first witnesses to seize the critical window of rescue, provide initial assistance to the patient, and thereby save lives, improve the patient’s condition, and alleviate their suffering (1, 2). These essential interventions not only buy valuable time for healthcare professionals before their arrival but also significantly enhance the patient’s quality of life (3, 4). In the current context, prioritizing “life first” stands as the core principle of rescue operations, representing the noblest value system and offering paramount social benefits (5). Given the escalating focus on health concerns and the evolving landscape of first aid practices, the establishment of a comprehensive first aid system has emerged as a societal consensus. It is imperative that all citizens receive first-aid education and acquire fundamental first-aid skills (6).

Various emergencies necessitate first aid, including but not limited to situations such as cardiac arrest, asphyxiation, and fractures (7). Among these, cardiac arrest is a prevalent and highly lethal global public health issue (8). Cardiac arrest occurs when the heart ceases its normal mechanical activity, leading to a lack of oxygen and nutrients in the brain and other organs, thereby posing a life-threatening risk to the patient (9). Consequently, cardiopulmonary resuscitation (CPR) has consistently been regarded as one of the most crucial first aid skills (10). In recent years, the incidence of sudden cardiac arrest worldwide has risen due to the impact of epidemics and unhealthy lifestyles. Statistics indicate that approximately 500,000 people die from cardiac arrest in the United States annually (11), 300,000 people experience out-of-hospital cardiac arrest in Europe each year (12), and around 1,030,000 patients suffer from out-of-hospital cardiac arrest in China annually (13). If cardiac arrest patients are deprived of oxygen for more than 4 min, they may experience irreversible brain damage or even death (14). Hence, it is imperative for these patients to receive appropriate first aid measures before reaching the hospital. Despite the growing awareness of the importance of first aid education, surveys reveal a significant gap between people’s willingness to learn first aid knowledge and skills and the actual training received (15). Currently, the prevalence rate of first aid knowledge in China is less than 1% (16), only 17.0% of the public is trained in performing CPR, and the success rate of out-of-hospital CPR is below 4.5% (17). This alarming situation highlights deficiencies in the public first aid education system.

First aid education comprises both theoretical and practical components (18). Assessments of first aid skills often focus on theoretical knowledge, practical skills, or a combination of both (19). In China, mainstream public first aid education models can be categorized into two main types. The first is indirect popularization, which involves disseminating scientific knowledge through media channels like newspapers, magazines, television, and the Internet. While this approach effectively spreads theoretical knowledge, it does not significantly enhance the public’s first aid skills (20). The second approach is direct popularization of science, where trainers interact face-to-face with the public through methods such as lectures, group training, and simulation exercises. While this method enhances both knowledge and skills, its effectiveness depends on the availability of adequate facilities and often requires substantial investment in teaching aids (21). Lectures, similar to indirect popularization of science, primarily focus on theoretical explanations. The quality of learning depends on the content and the trainer’s expertise. Purely theoretical teaching can lead to disengagement among the audience (22). Experts recommend incorporating more interactive elements, discussions, and hands-on training into first aid education to enhance its appeal (23, 24). Hands-on training, involving theoretical explanations, skill practice, and assessments, fosters active learning and engagement. However, public access to first aid training resources is limited, often requiring self-funding. This limitation dampens public enthusiasm for first aid training (25). Moreover, first aid knowledge acquisition is a gradual process. It is challenging for trainees to attain and retain skills through a single training session (5). Traditional first aid popularization models are often temporary campaigns or one-time basic training, lacking continuous education. This approach fails to meet the public’s learning needs and maintain their first aid proficiency over time. Consequently, there is an urgent need to reform the traditional first aid education model.

Public first aid education, designed to cater to the general public, must meticulously consider the learning needs and the overall learning experience of individuals in its teaching model. User experience (UX) encompasses users’ behavior, emotions, and their satisfaction level when interacting with a product or system (26). Psychologists have discovered that human behavior is significantly influenced by emotions (27). Emotions can be broadly categorized into positive and negative, with humans naturally inclined toward positive emotions and avoiding negative ones (28, 29). Positive emotions can enhance learning interests and foster proactive learning behaviors (30). Consequently, the concept of user experience has been integrated into educational design to create more engaging learning experiences and meet students’ requirements. Despite this, limited research has thoroughly examined user experience details during participants’ engagement in public first aid education or optimized the first aid education model based on these findings.

In light of this background, this study aims to delve into the current factors influencing the public’s acceptance of first aid education and the quality of learning, focusing on the user experience perspective. The study seeks to design a public first aid education model that aligns with users’ needs. We conducted user interviews to comprehend users’ behavioral motives, perceptions, and requirements throughout their participation in first aid learning. These insights serve as the foundation for crafting a new first aid education model. Using CPR as an example, the study encompasses both software and hardware design of the new education system. Finally, the program’s viability is assessed through user satisfaction evaluations. This research seeks to validate the applicability of the user experience design method in creating a public first aid education model and offers a valuable case reference for optimizing and reforming public first aid education programs.

## 2 Literature review

### 2.1 Public first aid education

Emergency medicine has historical roots in wartime practices, where the core objective has always been to minimize human casualties. In 1878, the Knights of St. John pioneered first aid training and ambulance services at public events in the United Kingdom, sparking widespread civilian interest in first aid (31). Over time, public first aid has gained recognition as an indispensable component of public health and emergency preparedness (32). With the global rise in disasters, epidemics, and injuries, empowering individual citizens with immediate response skills has become paramount (33). First aid education imparts life-saving techniques such as cardiopulmonary resuscitation (CPR), hemorrhage control, use of automated external defibrillators (AED), and methods for relieving choking or allergic reactions (34). Furthermore, it raises awareness about situations and behaviors that can cause or exacerbate health issues, promoting injury and illness prevention. By enhancing the community’s medical resources, public first aid education prevents complications and even saves lives before specialized medical assistance arrives (35, 36). It transforms citizens into active partners in emergency response efforts, thereby promoting public health equity and benefiting society as a whole.

Governments worldwide are taking proactive measures to promote first aid education among their citizens. Initiatives include integrating first aid training programs into school curricula (37), mandating first aid certificates for individuals in specific specialized positions, and funding or offering free first aid courses to residents. However, the development of public health services varies significantly across regions, leading to disparities in resources for public first aid education. Economic, governmental, geographic, social, and cultural factors contribute to this imbalance globally. Providing effective first aid education services to a broader population remains an ongoing challenge.

### 2.2 Public first aid model

Technical standards for public first aid are established by government agencies and certification organizations (5), while educational models are increasingly diverse and flexible. Regions and organizations can now choose the most suitable model based on their specific needs and circumstances (38). It is widely recognized that traditional educational models often suffer from limitations such as insufficient resources and a rigid, one-size-fits-all format.

To address the gaps in existing first aid education resources, communities have begun exploring innovative public first aid training models. Some scholars advocate providing regular first aid courses tailored for highly educated individuals with strong learning capabilities (39). Additionally, targeted CPR training is suggested for the older adults and family members of patients with cardiovascular diseases, given their higher risk of experiencing cardiac arrest (40, 41). These strategies aim to enhance the quality of education and increase the adoption rate of first aid techniques.

Educational concepts have evolved, leading scholars to explore pedagogical approaches like the peer learning model. This method focuses on increasing classroom interactions and participant engagement, overcoming the limitations of traditional educational models (42, 43). Moreover, many experts advocate the use of a blended learning model in public first aid education. This approach allows participants to learn online before engaging in face-to-face sessions. Blended learning combines online and offline teaching methods, expanding the learning format and boosting participants’ confidence and motivation (44, 45). The blended learning model is poised to become a significant trend in public first aid education, offering a more comprehensive and effective learning experience for participants in the future.

### 2.3 Public first aid tool

First aid education goes beyond mere theory; it necessitates essential supporting facilities and teaching tools. Effective tools can significantly enhance the learning experience and the overall quality of education. Scholars have proposed innovative approaches to enhance public participation in first aid learning. For instance, setting up first aid safe houses in communities provides residents with free access to first aid training venues, necessary hardware facilities, and volunteer guidance, thereby encouraging more people to engage in first aid education (46, 47).

Serious games have emerged as valuable tools in health education, effectively reducing resistance during first-aid training. Their entertaining nature boosts children and young people’s interest, actively engaging them in the learning process (48, 49). Music is another excellent supplementary educational tool. To simplify first aid knowledge and make learning enjoyable, experts have transformed first aid knowledge into songs and square dances. These familiar forms capture public attention and participation, making learning more accessible (50). Moreover, during CPR training, music or metronomes are recommended to help trainees maintain a stable chest compression rate, enhancing training effectiveness and enjoyment (51, 52).

In recent years, technological advancements have revolutionized learning methods. The rise of short-video culture and the availability of online platforms, such as short videos and microclasses, enable flexible, location-independent learning. Researchers have delved into short-video production elements to enhance user attention, content absorption, and learning willingness, contributing to the widespread adoption of health education through short videos (53, 54). Additionally, experts have advocated the use of virtual simulation systems for first aid technology exercises and high-fidelity mannequins with audio-visual feedback devices for real-time training guidance, enhancing the quality of training (55–57).

However, current evaluations of educational models and tools predominantly focus on knowledge and skill acquisition, overlooking the trainees’ experiential aspects. It is imperative to consider and assess the learners’ experience with these models and tools, ensuring a holistic approach to first aid education.

### 2.4 User experience design and education

Indeed, education can be viewed both as a process and a product (58). According to Don Norman, the relationship between a product and its users is dynamic, multifaceted, emotional, and perceptual. He advocates for a human-centered approach to product design research, making human-centeredness the core of user experience (UX) design (59). UX design involves a user-centered design research process, focusing on understanding users’ needs, behaviors, and perceptions to ensure that the product aligns with users’ requirements (60). Consequently, user research becomes a critical component of UX design, involving in-depth studies of target users and relevant stakeholders to comprehend their needs and preferences, guiding essential design decisions. Although there is not a fixed standard for the UX design process, it generally includes research and analysis, information architecture construction, prototyping, and user feedback testing.

Presently, UX design principles have been widely integrated into educational design, aiming to provide users with more engaging, effective, and personalized learning experiences. This application can be seen in UX-based online learning tutoring platform design (61), as well as in educational game design (62), among others. Given these successful applications in the realm of education, it can be inferred that a UX-based design approach for first aid education models is not only effective but also feasible. By incorporating human-centered principles into first aid education models, it is possible to enhance the learning experience, making it more engaging, effective, and tailored to the specific needs of the learners.

## 3 Methods

**Figure 1:**
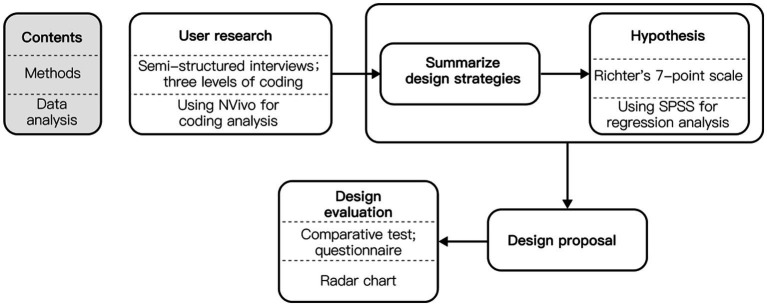
Research process and methods.

This study adopts the User Experience (UX) design methodology. Initially, in-depth research on target users was conducted through semi-structured interviews. The interview results were subjected to three-level coding analysis using Nvivo software. This method involves hierarchical classification and organization of information and is commonly used in qualitative research. It helps in organizing complex textual data effectively. In the second step, a design strategy was formulated based on the outcomes of the coding analysis. For uncertain aspects of the strategy, hypotheses were generated and studied. Moving to the third step, a design solution was proposed, grounded in the previously developed design strategy. Finally, users were invited to evaluate the new educational model. The detailed research process and the corresponding methods employed are illustrated in Figure 1.

### 3.1 User research

Offline training can simultaneously improve the public’s first aid knowledge and skill level, which is a more effective and already widely used first aid education model. Between April and May 2023, the researcher participated in four offline first aid education programs. The events were held at one community service center, one citizen emergency rescue center in Guangzhou, China, and two first aid centers in Shenzhen, China. The participants were recruited on-site at the above event locations.

To ensure a diverse and representative sample, certain groups, such as medical students and industry workers with a potentially biased perspective due to their specialized knowledge of emergency medicine, were excluded from the participant recruitment process. Efforts were made to invite participants of varying genders, ages, and trades, aiming for a broad spectrum of perspectives. This approach ensured a comprehensive understanding of first aid education experiences among different demographics. It is worth noting that the study extended its focus beyond just the trainees. Acknowledging the crucial role of trainers and their assistants as knowledge disseminators in first aid education campaigns, equal attention was given to interviewing both trainees and staff members. In total, six trainees and four staff members (trainers and their assistants) who participated in the events were interviewed for this study. Specific participant characteristics are detailed in Table 1.

**Table 1:** Characteristics of the participants.

Personnel type	Coding	Gender	Age	Level of education	Job	Interview location
Trainees	P1	Female	42	Undergraduate	Salesperson	Shenzhen^1^
	P2	Male	33	College	Engineer	Shenzhen^1^
	P3	Female	19	College	Student	Shenzhen^1^
	P4	Female	48	Undergraduate	Housewife	Guangzhou^2^
	P5	Male	51	Undergraduate	worker	Guangzhou^2^
	P6	Male	25	College	white collar	Guangzhou^3^
Staffs	P7	Male	38	Undergraduate	First Aid Educator	Shenzhen^1^
	P8	Female	29	College	First Aid Educator	Shenzhen^1^
	P9	Female	41	Undergraduate	Community Director	Guangzhou^2^
	P10	Male	24	College	Rescue team members	Guangzhou^3^

^1^First aid center.^2^Community service center.^3^Citizens Emergency Rescue Center.

### 3.2 Research approach

For the interviews, semi-structured outlines were meticulously prepared for both groups of research subjects. The interview questions were carefully designed to align with the flow of the activity, aiming to comprehensively understand the specific behaviors and perceptions of the interviewees before, during, and after the activity. Additionally, the questions delved into their perspectives on the current first aid education model. The fundamental questions’ content is detailed in Table 2.

**Table 2:** Interview outline.

Personnel type	Interview outline
Trainees	Why did you decide to attend this emergency science activity? How did you become aware of the existence of this activity? Can you describe your overall experience and understanding of the process while participating in this activity? Which part is the most satisfied/dissatisfied? Have you acquired knowledge in first aid from other sources? How effective was that learning experience? Through what channels/methods to you hope to master first aid knowledge and skills? Would you like to share this activity with your family and friends? After participating in this activity, are you willing to assist others in case of emergencies?
Staffs	how do you notify the public of these activities? How do you typically present your knowledge and skills to the audience? What is your evaluation of the entire educational process? How do you feel about your teaching? Which part is the most satisfied/dissatisfied? What do you believe are the limitations of the current first aid education model?

During the interviews, the researcher posed appropriate follow-up questions based on the participants’ responses. These follow-ups were tailored to guide interviewees into expressing more detailed feelings and opinions about their experiences. The interviews were conducted face-to-face, allowing for in-depth discussions that lasted between 15 to 30 min. With the participants’ consent, the entire conversation was recorded. Subsequently, the audio content was transcribed into textual information using speech recognition software (Xunfei Hear). The transcriptions were then meticulously analyzed using NVivo 11, employing a three-level coding methodology to extract meaningful insights from the data.

### 3.3 Data analysis

The analysis of the interview texts followed a systematic approach. Initially, the texts were carefully examined sentence by sentence. Expressions conveying similar meanings were consolidated and coded into 53 initial concepts. This initial coding aimed to define the concepts as well as the attributes identified in the interview text. Considering that user sentiment plays a pivotal role in understanding the user experience, emotional identification (positive, negative, or neutral) of the initial concepts was integrated into the coding process. This emotional categorization was accomplished using emotion vocabulary present in the text. In instances where explicit emotion vocabulary was absent, categorization was performed in relation to the context and the emotional tendencies evident in the text. If it was not feasible to distinctly categorize the sentiment, the emotion was classified as a neutral attitude. The entire initial coding process is meticulously documented in Table 3, with annotations indicating the sentiment categorization for each code.

**Table 3:** Initial coding process (a part).

Initial concept	Representative original statement
Prevent accidents	The old people in my family are old and not in good health, and I’m afraid of any emergencies ...... Learning some first aid skills can be a precautionary measure.
Social platform	I read about the activity on WeChat.
Sufficient practice time^1^	Everyone gets 5 chances to practice ...... I even went to practice at halftime!
Interesting^1^	For the first time in my life, I knew I could remember to press the beat with this song, and it’s pretty fun!
Training takes too long^2^	The training was too long ...... the whole day has been spent here
Afraid of being blackmailed^2^	I’m afraid I’ll be blackmailed if I do not succeed in saving someone’s life.

^1^Positive emotions.^2^Negative emotions.

In the subsequent stage, the initial concepts derived from the interviews were systematically organized using clustering and merging techniques. Through several rounds of sorting analyses, 16 distinct initial categories were identified. These categories represented common themes found within the initial concepts. Following this, the interrelationships between these 16 initial categories were thoroughly examined. By discerning similarities, causal connections, and process relationships among these categories, more comprehensive main categories were formulated.

After rigorous analysis, five main categories emerged, capturing the essential themes and patterns inherent in the interview data. The initial categories and the corresponding initial concepts within each main category were meticulously documented in Table 4, offering a detailed overview of the thematic organization derived from the interviews.

**Table 4:** Three-level coding of interview materials.

Main category	Initial category	Initial concept
Behavioral motives	Learning motivation	Prevent accidents; leen for knowledge; finish the task; realize personal values
	Access to information	Social platform; recommendations from friends; meet offline
Objective demand	Learning location	Place of study; community; emergency center; school; companies
	Learning process	Explain the theory; demonstration; practice; movement guidance; examination; discussion
	Learning Points	Basic theory; sufficient practice; review regularly
	Functions of teaching aids	Practice simulation^1^; data logging^1^; feedback^1^
	Use of teaching aids	Sufficient practice time^1^; insufficient practice time^2^
Content perception	Teaching content	Simple content^1^; Complex content^2^; full content^1^
	Teaching style	Interesting^1^; active^1^; teaching too fast/slow^2^
	Appearance of teaching aids	Broken^2^; realistic form^1^
Cost perception	Cost of money	Free^1^; paid^2^; wear and tear on teaching aids^2^
	Cost of time	Training takes too long^2^; no time^2^; Activity location is far away^2^; activity location is close^1^
	Cost of labor	Inability to instruct all students at the same time^2^; handling of teaching aids^2^; sterilization of teaching aids^2^
Risk perception	Reluctance to learn	Fear of being too difficult to learn^2^; not knowing where to find effective learning resources^2^; specialized personnel for specialized tasks^2^
	Interruption of learning	Dirty teaching aids^2^; horrible appearance of teaching aids^2^; under the scrutiny of multiple individuals^2^
	Reluctance to use first aid skills	inflict secondary injuries on the patient^2^; Afraid of being blackmailed^2^; forgetting of knowledge^2^

^1^Positive emotions.^2^Negative emotions.

**Figure 2:**
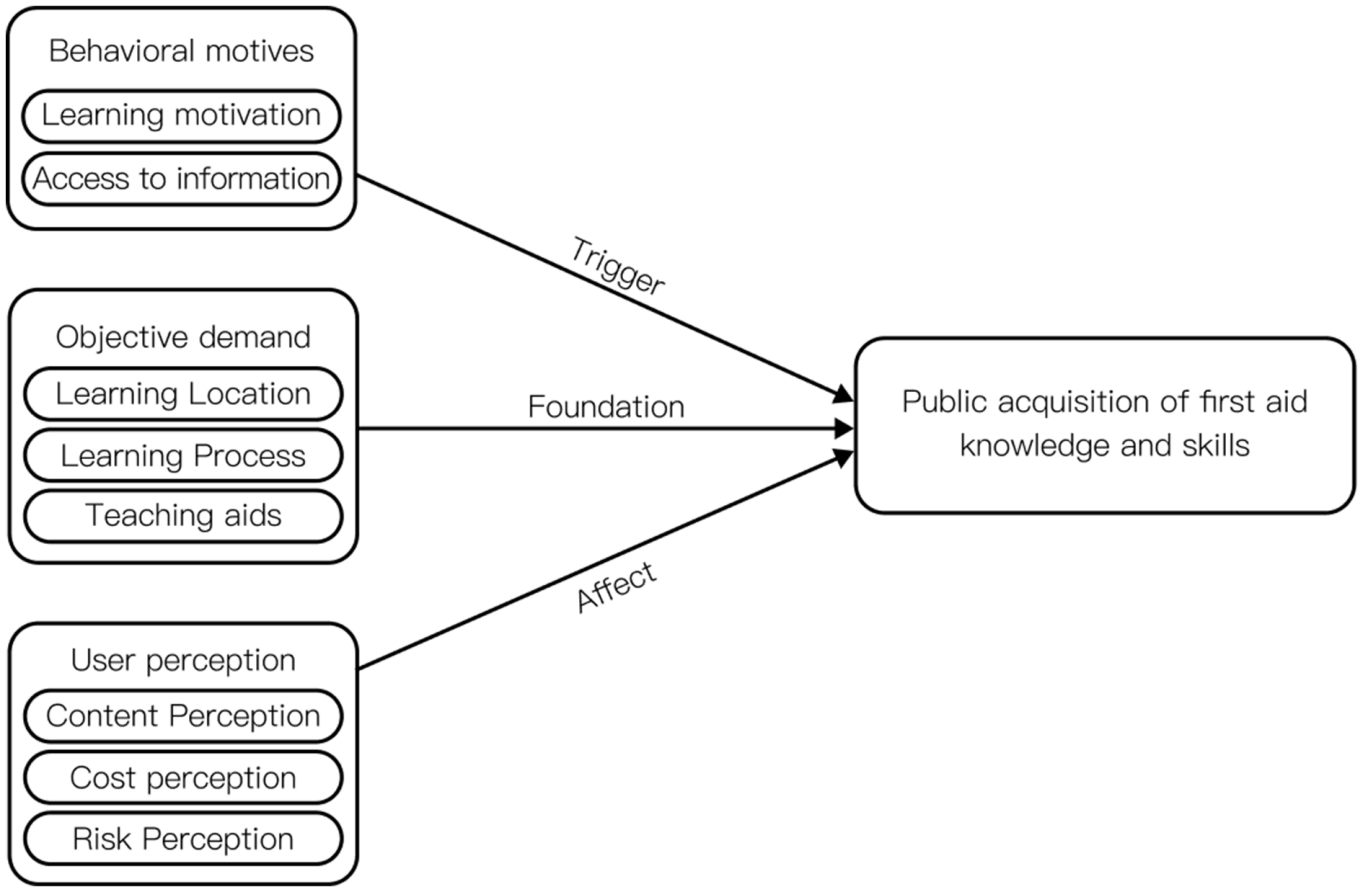
Factors influencing the public’s acquisition of first aid knowledge and skills.

Through the systematic categorization and logical organization of textual information and codes at all levels, a clear connection between the main categories and the design objective – “helping the public to acquire knowledge and skills in first aid” – was established, as illustrated in Figure 2.

“Behavioral motivation” pertains to users receiving external information stimuli that generate awareness of first aid learning. Subsequently, they actively or passively seek channels to acquire education. This awareness is a prerequisite for users to participate in first aid learning.

“Objective demand” encompasses the indispensable processes and material conditions necessary to complete first aid learning. These fundamental elements form the basis for successfully engaging in first aid education.

“User perception” relates to the subjective evaluation of the elements users encounter during the learning process. It includes emotional biases and individual perspectives. For instance, within the same first aid training activity, one participant might feel the teacher’s lecture speed is too fast (P1), while another might find it too slow (P2). Research has demonstrated that emotions significantly influence decision-making. Positive emotions enhance users’ favorable perceptions of a product, encouraging active interaction and improving understanding and memory (63). Therefore, the emotional component of “perception” significantly impacts the quality of learning. Increasing the proportion of positive emotions perceived by users during the learning process can enhance the overall quality of learning experiences.

Following this comprehensive analysis, this study identifies behavioral motivation, objective needs, and user perception as the core categories of the public first aid education model. These categories form the foundation for developing an effective and user-centered approach to first aid education.

### 3.4 Design strategies

**Figure 3:**
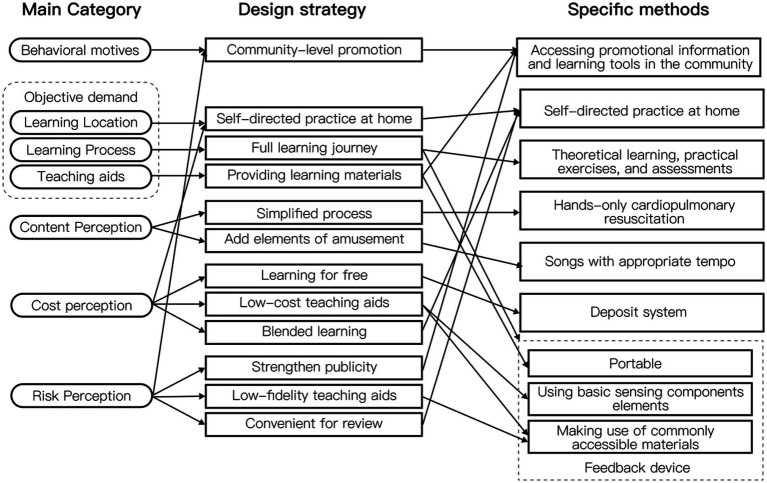
Design strategies and specific methods.

The design strategy presented in this study is firmly rooted in the principles of user experience. Drawing from the comprehensive results of the coding analysis in user research, a total of 12 design strategies have been meticulously formulated, as depicted in Figure 3. Central to this strategy is the principle of reinforcing factors that can evoke positive emotions while concurrently minimizing situations that might trigger negative emotions among users. This foundational approach underpins the logic behind the proposed design strategies, ensuring a user-centered and emotionally resonant first aid education model.

The proposed design, using CPR learning as an example, integrates findings from extensive literature research and user research to introduce specific solutions:

1. Community-Based Educational Activities: Implement educational activities within the community setting, utilizing locations like residential neighborhoods, schools, and parks as hubs for learning (64).
2. Independent Practice at Home: Encourage users to practice independently at home, allowing them to tailor their study time and duration according to their schedule, enhancing convenience in both learning and review.
3. Structured Learning Process: Design the learning process to include theoretical learning, practical exercises, and assessments, ensuring a comprehensive educational experience.
4. Hands-Only CPR: Promote the use of Hands-Only CPR, focusing on chest compressions only. Studies have indicated that this approach maintains similar survival rates to conventional CPR while being easier to learn and master, thus increasing public awareness and proficiency in basic first aid skills (1).
5. Incorporate Rhythmic Songs: Utilize songs with appropriate rhythms to assist users in controlling the compression rhythm during CPR. Music as an auditory sensory cue can enhance the level of immersion in the learning process (65). Additionally, studies have confirmed its positive impact on first aid training interventions (51, 52).
6. Implement a Deposit System: Introduce a deposit system for teaching aids. Users, after mastering the compression technique, may temporarily return the aids. Implementing a rental system increases aid reuse, promoting cost-effectiveness and environmental sustainability.
7. Design Requirements for Teaching Aids: Focus on essential design aspects for teaching aids, especially chest compression frequency and depth, crucial indicators of compression quality. Utilize basic electronic sensing elements to measure these parameters. Additionally, instead of highly realistic simulators that might induce fear, employ everyday objects simulating chest thickness and rebound, combining them into a simple, low-simulation feedback device. This approach maintains effectiveness while reducing costs and minimizing psychological pressure on users (66, 67).

## 4 Study hypothesis

### 4.1 Hypothesis

The use of music in CPR training is well-documented, with notable examples like the British Heart Foundation’s utilization of the song “Stayin’ Alive” in public CPR tutorials, which has been widely adopted in first aid training globally. Similarly, organizations such as the China Cardiovascular Health Alliance and the China Chest Pain Center Alliance have curated CPR first aid training song lists, incorporating familiar tunes like “The Most Dazzling National Wind” and “Calories.” While there are numerous songs suitable for first aid training, the specific impact of different songs on user learning effectiveness remains unclear. This ambiguity forms the basis for the first set of hypotheses:

*H1a:* Using familiar songs for learning compression rate, learners find it more engaging.

*H1b:* Using familiar songs for learning compression rate enhances learners’ motivation to learn.

*H1c:* Using familiar songs for learning compression rate enhances learners’ confidence to learn.

Furthermore, while the significance and feasibility of designing a simple feedback device have been elucidated in the design strategy proposal, the level of user acceptance regarding this low-simulation device remains uncertain. This uncertainty forms the basis for the second set of hypotheses:

*H2a:* Training with low-fidelity simulated feedback devices can reduce learners’ fear.

*H2b:* Training with low-fidelity simulated feedback devices increases learners’ motivation to learn.

*H2c:* Training with low-fidelity simulated feedback devices increases learners’ confidence to learn.

### 4.2 Questionnaire

Two separate questionnaires were designed to address the aforementioned hypotheses. Both questionnaires utilized a 7-point Likert scale, ranging from 1 (completely disagree) to 7 (completely agree).

Questionnaire 1 focused on evaluating participants’ responses to four songs with similar rhythm and chest compression frequency, aiming to understand the relationship between song familiarity and participants’ positive emotions. Additionally, this questionnaire investigated the connection between song familiarity and participants’ willingness to learn and confidence in learning.

**Figure 4:**
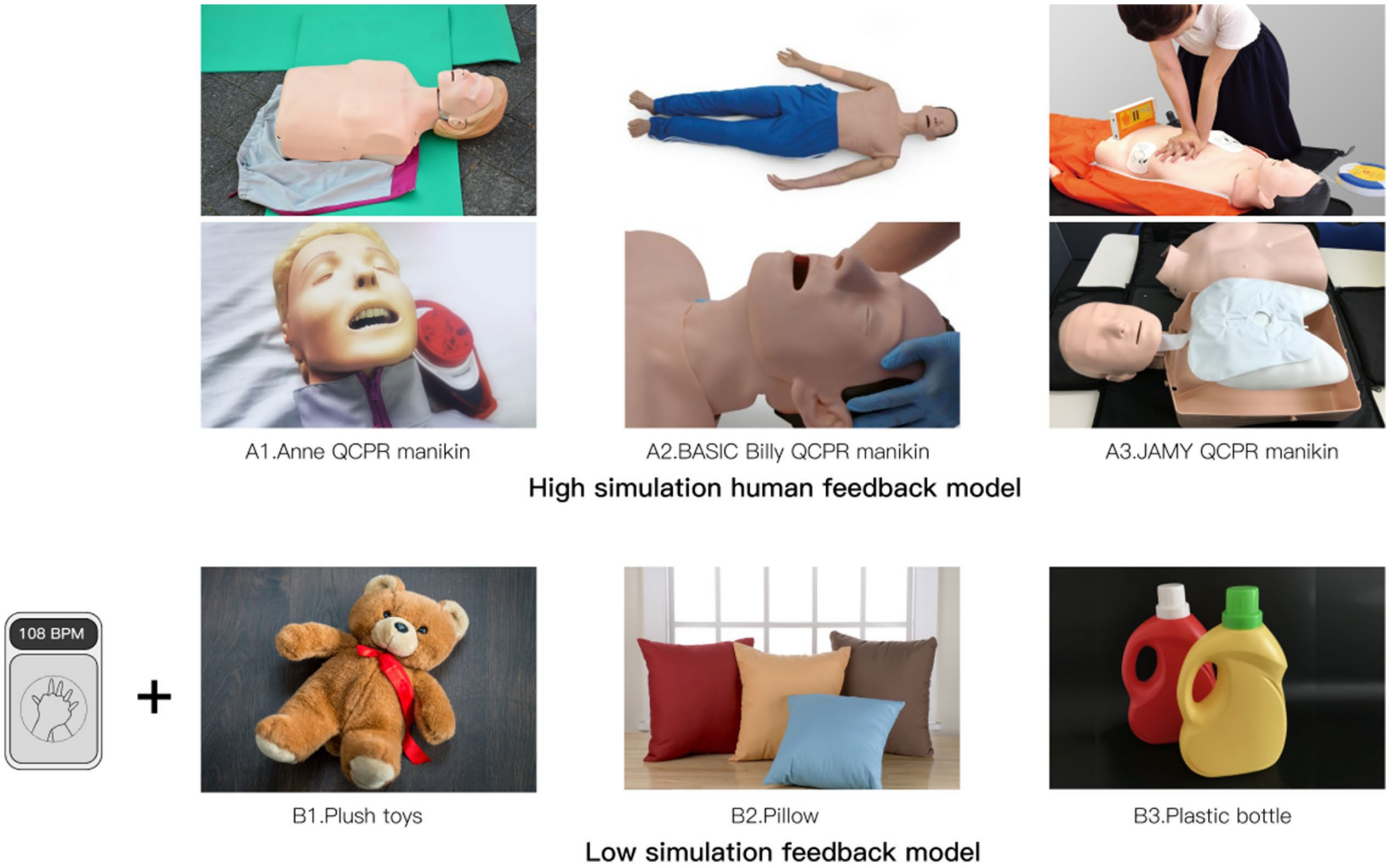
Questionnaire 2 material.

Questionnaire 2 presented participants with images of three high-simulation human feedback models and three low-simulation feedback models, as illustrated in Figure 4. The low-simulation feedback devices were created using a combination of small feedback devices and common household items such as stuffed animals, pillows, or empty plastic bottles. These devices measured depth and frequency during chest compressions. Participants were asked to rate their level of fear generated when viewing each model, their willingness to use it, and their trust in the model.

Both questionnaires included a demographic section at the end to collect additional participant information.

### 4.3 Participants

Participants, who lacked first aid training and medical expertise, were recruited through online social media platforms. Initially, 132 individuals completed the questionnaire. However, data from 10 participants were excluded due to poor questionnaire quality. Consequently, a total of 122 valid questionnaires were obtained for analysis, comprising 56 males and 66 females, with ages ranging from 18 to 60 years old. These participants were randomly assigned to complete one of the three questionnaires. Detailed demographic information for each group is presented in Table 5.

**Table 5:** Summary of participant demographics.

Demographic	Questionnaire 2(N = 61)	Questionnaire 3(N = 61)
Sex		
Male	26(42.6%)	30(49.2%)
Female	35(57.4%)	31(50.8%)
Age		
18–25	36(59.0%)	32(52.5%)
26–30	11(18.0%)	12(19.7%)
31–40	9(14.8%)	8(%)
41–50	2(3.3%)	6(%)
51–60	3(4.9%)	0(0%)
Education		
High school and below	6(9.8%)	13(21.3%)
College and Junior College	43(70.5%)	41(67.2%)
Graduate and above	12(19.7%)	7(11.5%)

### 4.4 Results

The questionnaire results were statistically analyzed using SPSS 27.0 software. A one-way linear regression analysis was conducted with song familiarity as the independent variable and the degree of fun perception as the dependent variable. The analysis demonstrated a highly significant positive effect of song familiarity on the degree of fun perception (B = 0.774, *p* < 0.001), confirming Hypothesis H1a.

Furthermore, the use of songs with high familiarity significantly and positively impacted participants’ willingness to learn (B = 0.309, *p* = 0.011 < 0.05) and confidence in learning (B = 0.346, *p* = 0.004 < 0.01), thus establishing Hypotheses H1b and H1c.

It is noteworthy that since all the participants were from China, their familiarity with local songs was higher than that with English songs. Consequently, the perceived level of fun, willingness to learn, and confidence in learning associated with local songs were also higher than those related to English songs, as illustrated in Table 6.

**Table 6:** Attitudes toward different song.

	English songs		Chinese songs	
	Stay in alive	Baby shark	Most dazzling national style	Calorie
Degree of familiarity	3.13	3.97	6.41	5.89
Degree of interest	4.10	4.70	5.84	5.49
Willingness to learn	3.93	4.61	5.69	5.34
Learning confidence	4.05	4.54	5.57	5.25

In the cross sectional comparison of participants’ mean fearfulness for each model (as shown in Table 6), it was evident that participants’ mean fearfulness of the low-simulation model was lower than that of the high-simulation model, confirming Hypothesis H2a.

Interestingly, despite the reduced negative emotions associated with the low-simulation feedback model’s appearance, participants’ willingness to use it was lower compared to the high-simulation model. However, when analyzed using a one-way linear regression model, the results indicated that fear had no significant effect on either willingness to learn (*p* = 0.474) or confidence in learning (*p* = 0.872). Therefore, Hypothesis H2b and Hypothesis H2c were not supported by the data (Table 7).

**Table 7:** Attitudes toward different model.

	High simulation human feedback model			Low simulation feedback model		
	BASIC Billy QCPR manikin	Anne QCPR manikin	JAMY QCPR manikin	Plush toys	Pillow	Plastic bottle
Degree of fear	4.41	4.39	3.74	2.52	2.10	2.26
Willingness to use	5.26	5.07	5.30	4.67	4.67	3.36
Confidence in the model	5.07	5.08	5.43	4.18	4.05	3.13

QCPR, Quality Cardioplumonary Resuscitation.

## 5 Design

### 5.1 Content frame

**Figure 5:**
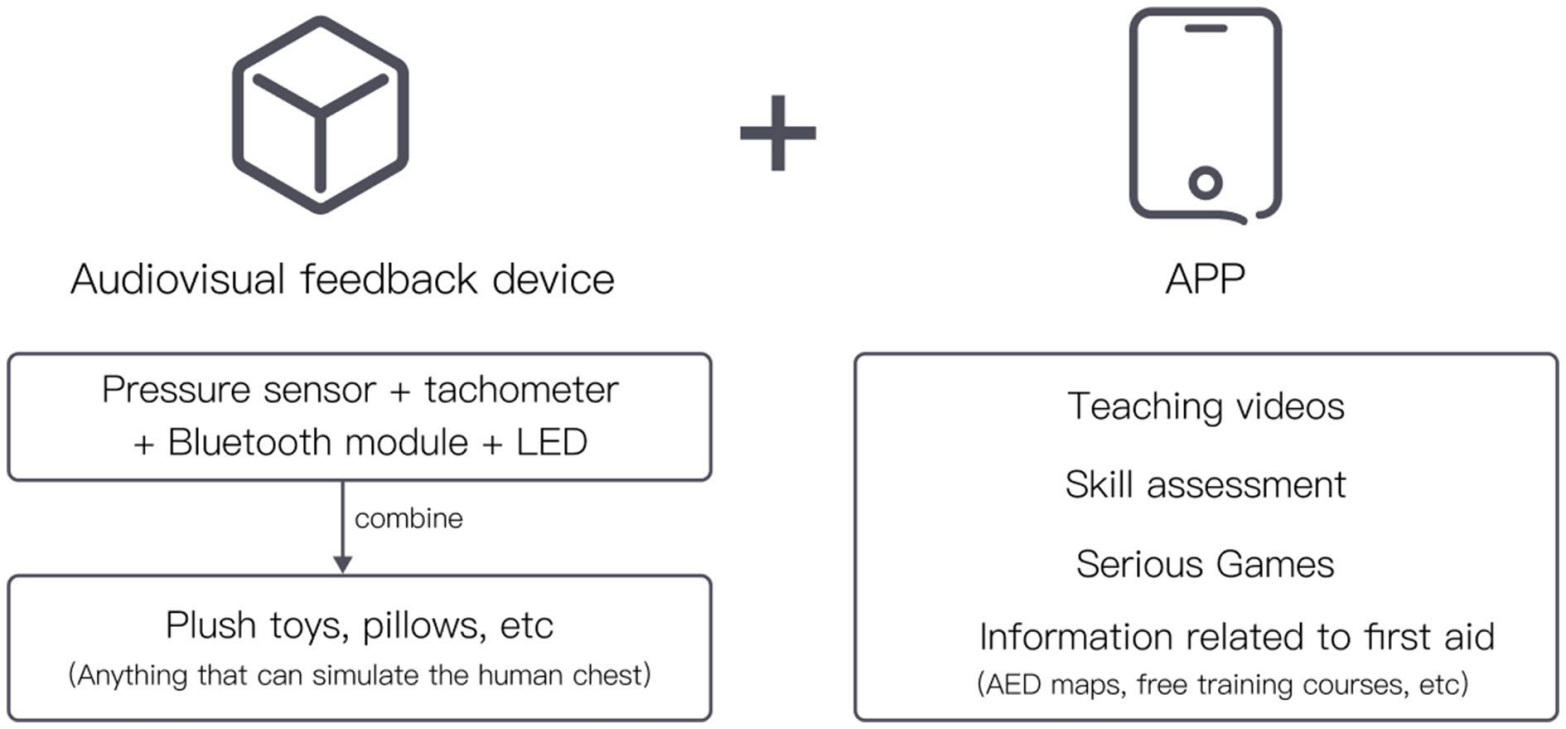
Framework.

The new public first aid education system is proposed in the format of a “feedback device + APP,” as depicted in Figure 5. Leveraging the widespread use of smartphones, incorporating certain product functions into an app can significantly decrease the development and production costs of the feedback device and enhance its accessibility. To distribute the feedback device, a self-service machine approach is chosen, allowing the public greater flexibility. Trainees can acquire the feedback device by scanning a code and submitting a deposit, ensuring easy access for those in need.

### 5.2 Design description

**Figure 6:**
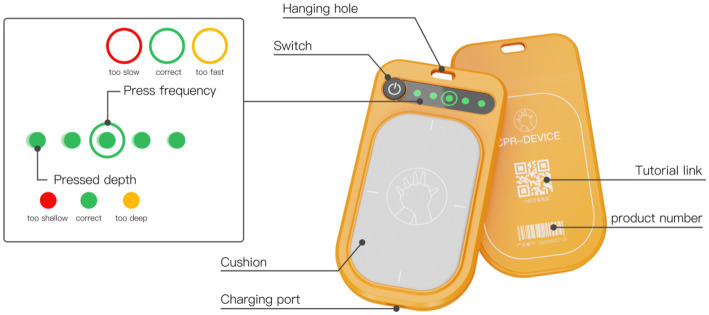
Concept design prototype and indicator light explanation.

The feedback device is equipped with a pressure sensor and a tachometer sensor, enabling it to calculate the depth and frequency of the user’s chest compressions. It provides real-time feedback to the user through the color of the light, guiding the user to adjust their actions promptly, as illustrated in Figure 6.

**Figure 7:**
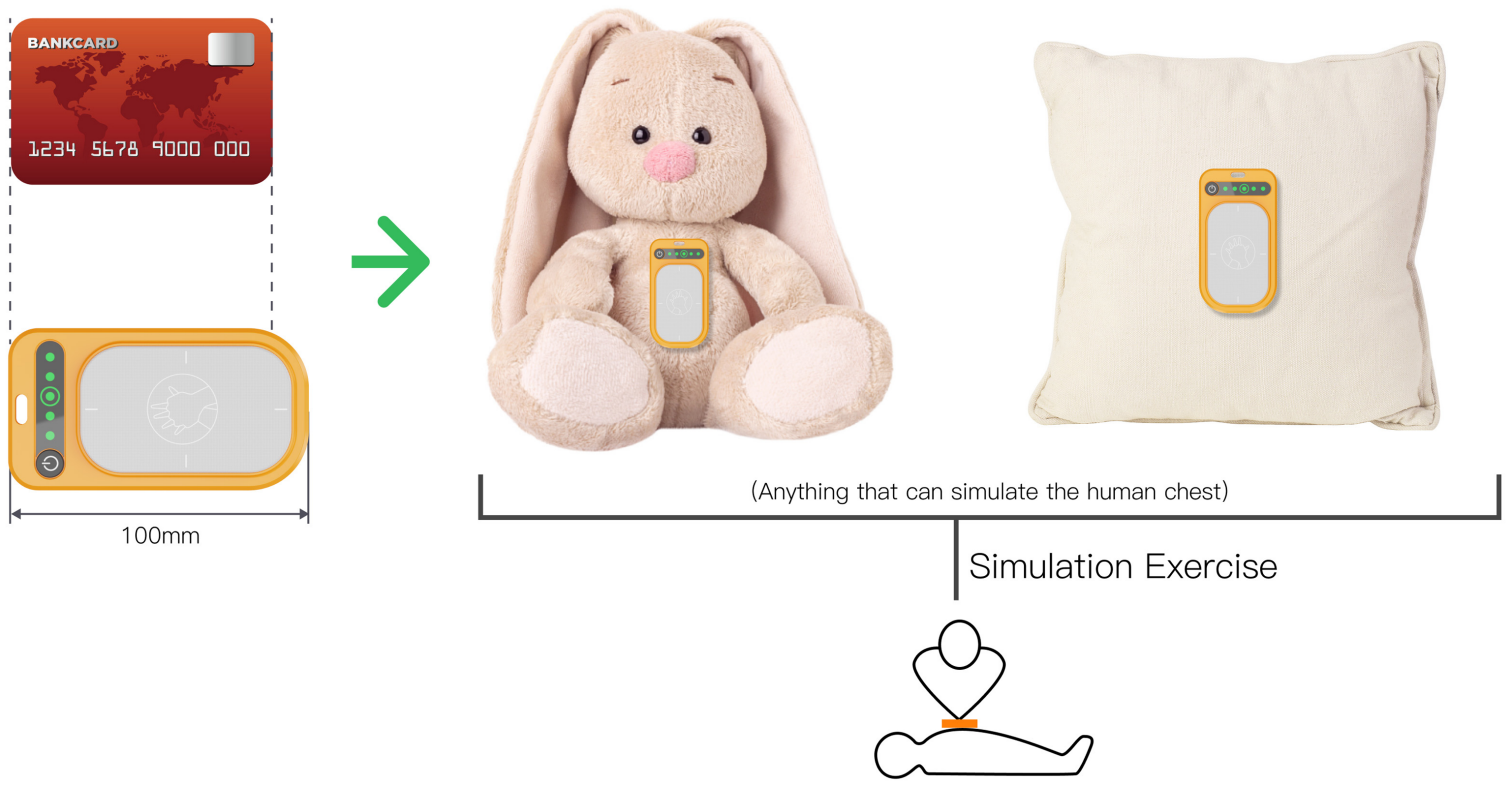
Product size and usage diagram.

The feedback device is designed to be compatible with various items such as dolls, pillows, and other objects that can simulate the human chest cavity. This setup forms a straightforward CPR practice device. The feedback device is compact, portable, and slender, resembling the dimensions of a credit card. This design allows users to conveniently carry it for practice and on-the-go use. During emergencies, the device can be placed on the lower part of the patient’s sternum, providing real-time guidance on the accuracy of chest compressions, as depicted in Figure 7.

**Figure 8:**
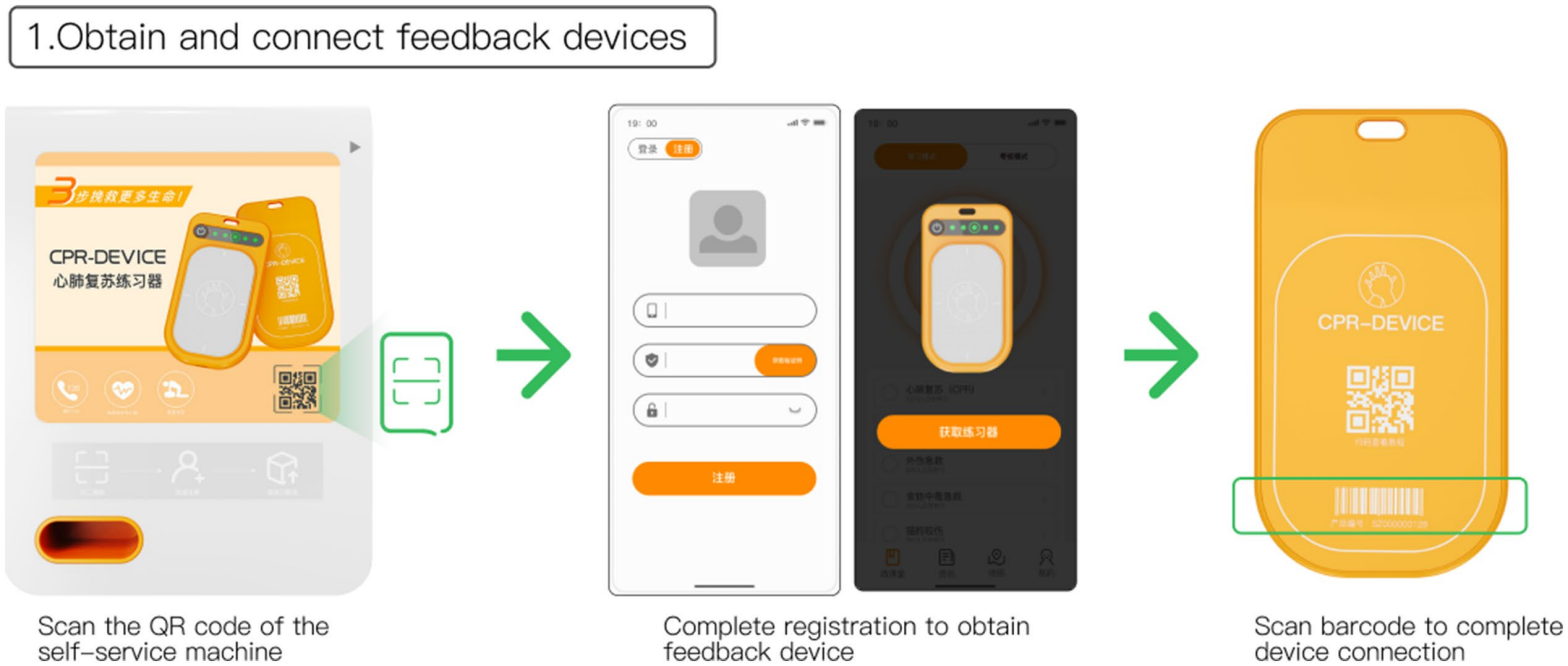
Obtain and connect feedback devices.

To enhance user convenience and accessibility, kiosks will be strategically placed in high-traffic areas within the community. Residents can obtain the feedback device through these community-sited kiosks following a simple process, as illustrated in Figure 8. Users will follow the instructions displayed on the kiosk, scan the QR code at the top of the kiosk using their smartphones, download and register on the dedicated app, request the feedback device, and submit the required deposit at the kiosk pickup port.

The feedback device comes with a built-in Bluetooth module, allowing users to quickly connect it to their personal account by scanning the barcode on the back of the device. This seamless connection enables real-time instruction through microclasses in the app during practice and allows users to review their exercise results afterward.

**Figure 9:**
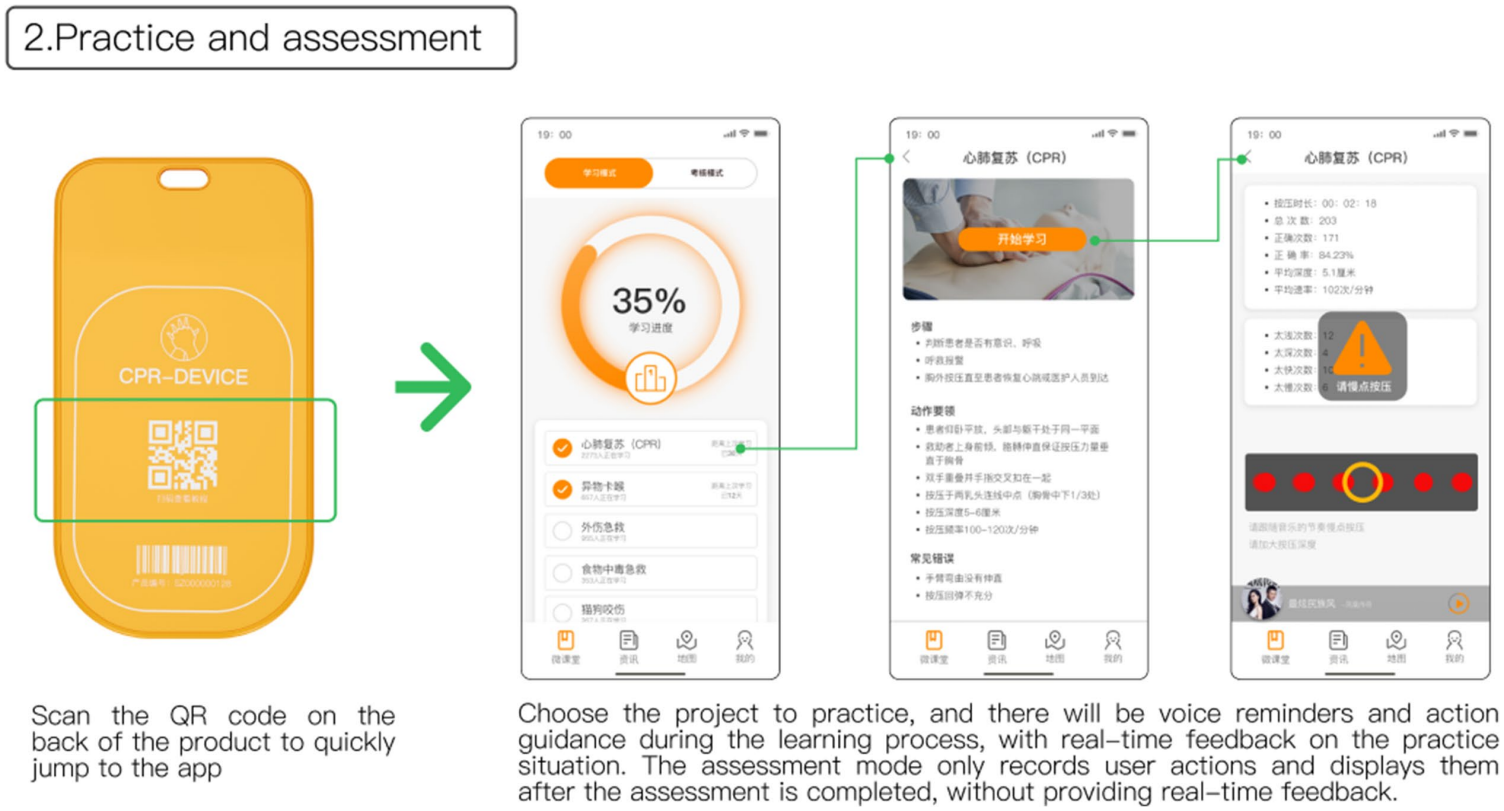
Practice and assessment.

Upon scanning the QR code on the back of the feedback device, users will be directed to the microclassroom interface within the app, as depicted in Figure 9. Here, users can choose specific CPR programs for learning. Throughout the learning process, the app provides voice prompts, real-time recording, and immediate feedback on the user’s practice. To enhance users’ understanding of the compression rhythm, the guidance process incorporates popular tracks like “The Most Dazzling National Style,” allowing users to master the chest compression rhythm through music.

The app includes an assessment mode to evaluate users’ knowledge and skills. In this mode, the device records the user’s actions without providing real-time guidance, allowing for a comprehensive assessment of the user’s proficiency.

**Figure 10:**
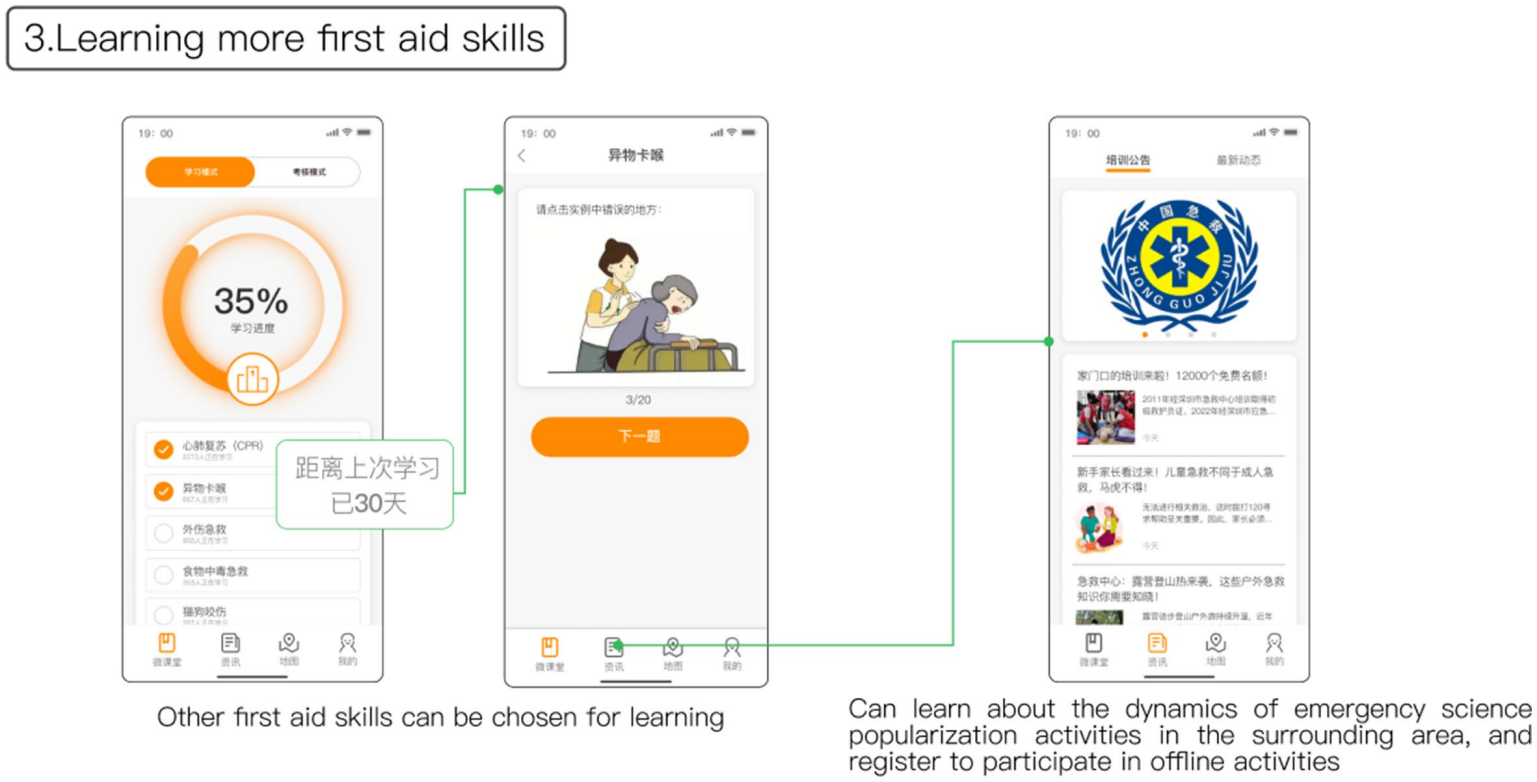
Learning more first aid skills and more information.

Figure 10 illustrates the microclassroom page, which features a progress status bar at the top to motivate users to complete various first aid skills. Users have the option to select different first aid skills for learning. Completed courses are marked with the time since the last completion, serving as a reminder for users to review regularly and reinforce their learning. To address the challenge of limited channels for promoting offline public first aid education activities, the information interface is utilized to showcase updates on community first aid skills training or awareness events. This allows users to easily access information about upcoming offline trainings or activities, enhancing their knowledge and professionalism in first aid skills.

**Figure 11:**
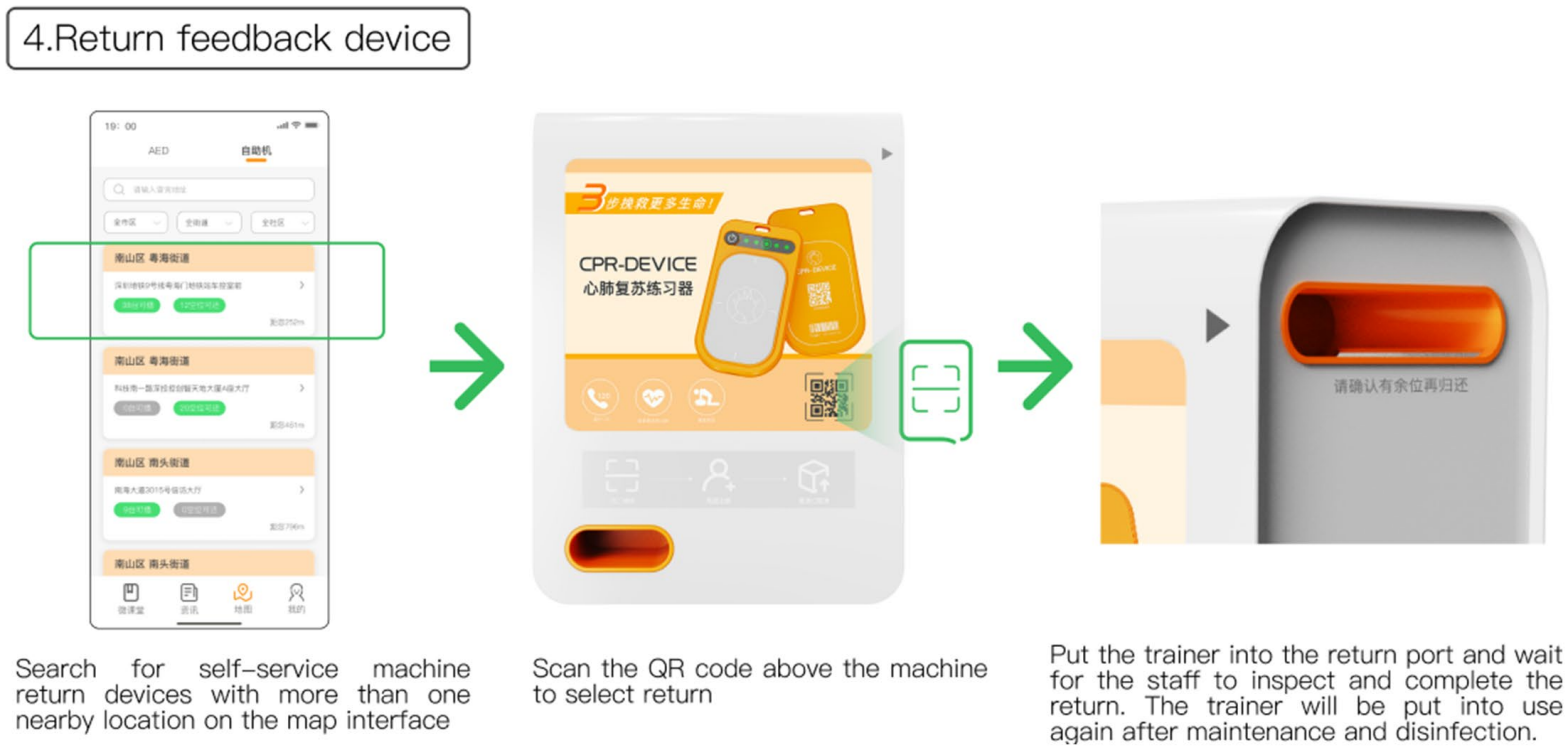
Return feedback device.

In the final step of the process, after completing the training, users can utilize the map interface to search for nearby kiosks with available slots to return the feedback device, as depicted in Figure 11. Upon reaching the designated kiosk, users can follow the on-screen instructions and place the feedback device into the return port situated on the upper right side of the kiosk, initiating the return audit process. Once the staff verifies the device and returns the deposit, the return process is finalized. Following maintenance and sterilization, the feedback device will be made available for use again. Users also have the option to retain the exerciser by default, essentially purchasing the product if they choose not to return it.

### 5.3 Design evaluation

In this stage, a structured questionnaire was utilized to gather user satisfaction evaluations of both the new first aid education model and the traditional first aid education model. The traditional training mode, in this context, involves community staff inviting professional first-aid training instructors to conduct first-aid knowledge lectures for community residents, followed by on-site first-aid skill demonstrations and drills. The questionnaire presented participants with detailed descriptions of both the new and traditional first aid education models and included a scale for evaluation. Participants rated their satisfaction on a scale ranging from very satisfied to very dissatisfied. The scale focused on five dimensions: the flow of the activity, the content of the activity, the time schedule, the teaching method, and the equipment used. Participants were randomly assigned either A or B volume of the questionnaire to fill out, with each volume covering one of the models.

Once participants completed the questionnaire, the average values for each item were calculated to assess satisfaction with the respective dimensions of both the new and traditional first aid education models.

### 5.4 Results

**Figure 12:**
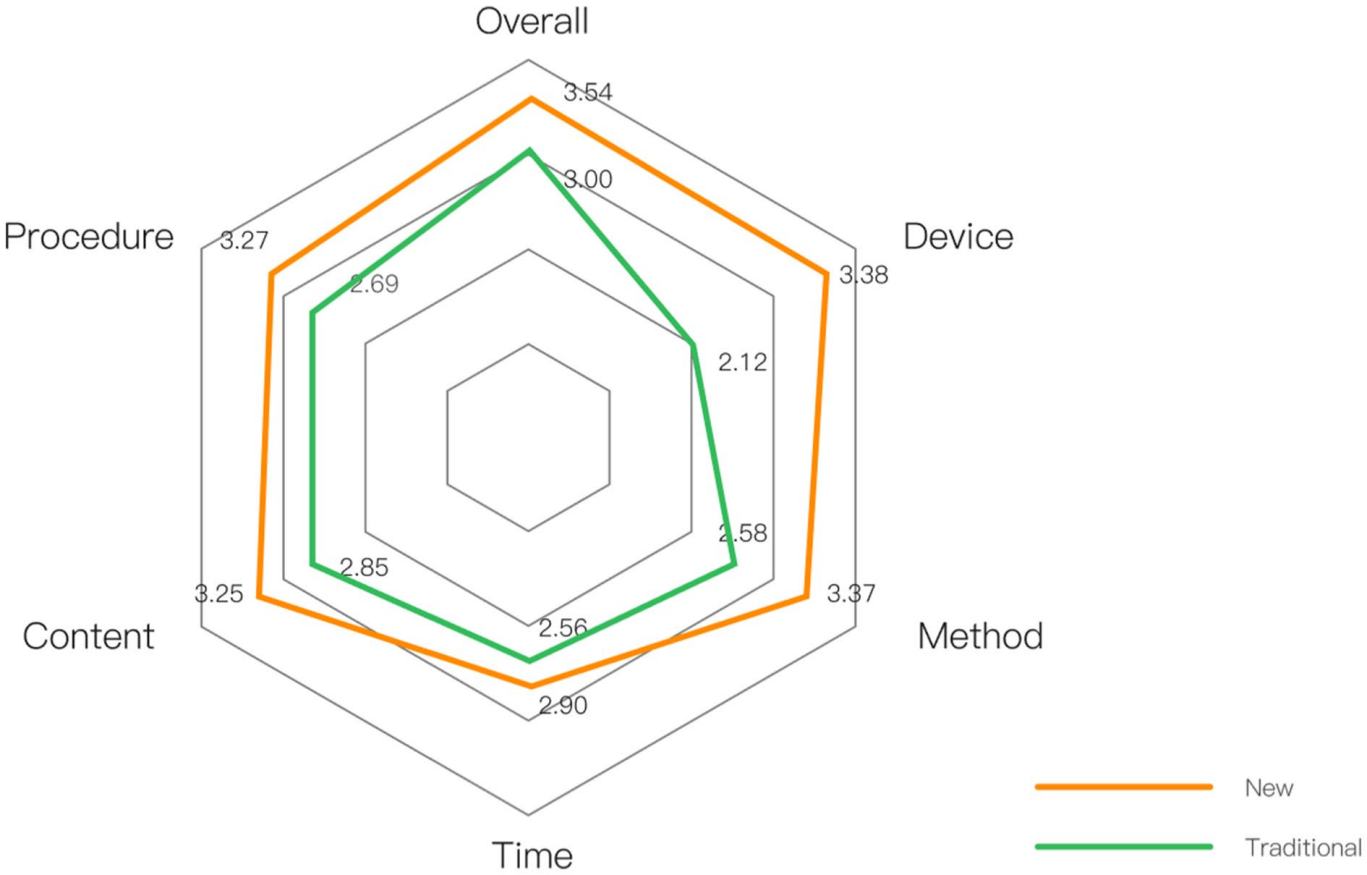
Comparison of satisfaction evaluation between new and traditional models.

The evaluation results from 52 valid questionnaires (23 males and 29 females, age 20–50) are displayed in Figure 12. The mean values indicate that users’ satisfaction with all dimensions of the new model surpassed those of the traditional model. Notably, in terms of equipment satisfaction, users expressed significantly higher satisfaction with the equipment used in the new model (Mean = 3.38 > Mean = 2.12), reflecting the positive reception of the new model among users.

## 6 Discussion

First of all, in order to fill the gap of user experience design in the application of public first aid education, this study designed an innovative first aid education model based on the results of user research, which is presented in the form of “feedback device + app.” Through user evaluation, we found that users’ satisfaction with this program was higher than that of the traditional model in all dimensions, thus proving the acceptability of the public first aid education model based on user experience design.

Second, based on user research, this study summarized the factors affecting the public’s acceptance of first aid training and the quality of training through three levels of coding. The purpose of public first aid education is to equip the public with first aid knowledge and skills. While receiving first aid training is only a behavioral process, it does not indicate that the user has fully mastered what was learned in the training. Therefore there is also a need to use the quality of training to measure the user’s mastery of knowledge and skills. Many researchers have also validated the feasibility of their studies by measuring and comparing users’ knowledge skill mastery (52, 56, 57).

Third, this study found that familiar songs induced more positive emotions and were more likely to increase users’ willingness to learn and confidence in learning. Positive emotions can promote one’s learning ability and performance (68). Previous studies have shown that music has a positive effect on CPR learning (51, 52). Since there are differences in individual familiarity with songs, users can choose songs with appropriate tempo among familiar songs to assist their memorization without forcing themselves to memorize unfamiliar tracks.

Fourth, this study found that users generated less negative emotions when facing the low-simulation model compared to the case of the high-simulation model, but at the same time, the willingness to use the low-simulation model was also lower, which is inconsistent with our expected results. The phenomenon may be attributed to a lower level of trust in low-simulation models, possibly due to perceived inadequacies in their appearance, tactile feedback, and overall professionalism compared to high-simulation models (69). However, it has been shown that for novices, only a small portion of the feedback is noticed by the user (70). Therefore, this study still insists on using a low-simulation model in the design scheme, which is more portable and less expensive than the high-simulation model, and is more suitable for wide-scale dissemination.

Due to cost considerations, the feedback device in this design solution only retains the calculation of chest compression frequency and depth, which cannot guide the trainee’s compression posture and position. Although it can be considered to capture and action through cell phone camera shooting and use artificial intelligence for auxiliary analysis (71), it is not yet able to completely replace the role of professional trainers for the time being. Therefore, this design solution is more suitable for self-training of trainees who are just starting out. Although it cannot completely replace offline first aid training, it can be used as pre-training to improve the public’s first aid awareness and performance in offline professional training (72), which coincides with the idea of blended learning (44, 45).

## 7 Limitations and further research directions

The study has several limitations that need to be addressed. Firstly, the selection of sampling locations was constrained by the researcher’s location during the user interview research phase, potentially introducing selection bias. To enhance the study’s generalizability, future research could explore sampling locations more comprehensively, ensuring a broader geographical representation.

Secondly, the design solution presented in this study remains at the conceptual stage and has not been prototyped. Consequently, participants’ understanding of the solution relies solely on the researcher’s description, lacking practical experience. This reliance on descriptions could lead to evaluations influenced by participants’ cognitive biases. To mitigate this limitation, it is essential to move beyond the conceptual phase. Researchers can create prototypes, engage participants to interact with the solution, and gather feedback based on their real experiences. Through iterative testing and refinement informed by user feedback, the product can be substantially improved.

Additionally, this study focused primarily on CPR skills, neglecting other crucial first aid techniques necessary for emergency situations such as choking, bleeding, and food poisoning. The paper did not explore how the feedback device and accompanying app could be utilized to practice these skills. Future research should delve into integrating other essential first aid skills with the strategies proposed in this study. By investigating ways to teach and assess various first aid skills within the framework of the proposed feedback system, the entire public first aid education system can be significantly enhanced.

In summary, addressing these limitations through comprehensive sampling, prototype development, real-user interaction, and the incorporation of diverse first aid skills will strengthen the validity, reliability, and practicality of the study’s findings, contributing significantly to the advancement of public first aid education.

## 8 Conclusion

This study introduces an innovative first aid education model grounded in user experience design methodology, utilizing a feedback device and an accompanying app to facilitate self-guided first aid learning. The model aims to enhance users’ learning experiences by fostering positive emotions and minimizing negative ones. Through user feedback, the educational model’s design concept gained user recognition. This research not only presents a novel approach to designing public first aid education but also serves as a valuable reference for program development, contributing to the ongoing reform in this field.

Moving forward, future research will concentrate on rigorous testing and evaluating the proposed design solution. This includes addressing challenges that users might encounter during application, ensuring the model meets users’ needs and expectations. Through iterative improvements and upgrades, the goal is to create a public first aid education model that aligns seamlessly with user requirements. Ultimately, this iterative process aims to significantly enhance the model’s popularity and implementation rate, specifically in the context of public CPR training.

## Data availability statement

The original contributions presented in the study are included in the article/supplementary material, further inquiries can be directed to the corresponding author.

## Author contributions

JL: Conceptualization, Funding acquisition, Investigation, Methodology, Project administration, Supervision, Writing – review & editing. KZ: Conceptualization, Data curation, Formal analysis, Software, Visualization, Writing – original draft. WH: Conceptualization, Methodology, Project administration, Resources, Validation, Writing – review & editing.
